# Cost-effective analysis of sugemalimab plus chemotherapy as first-line treatment for advanced gastric or gastroesophageal junction adenocarcinoma with PD-L1 CPS ≥5

**DOI:** 10.3389/fpubh.2025.1604372

**Published:** 2025-08-22

**Authors:** Yalan Zhang, Ying He, Ruijia Chen, Maojin You

**Affiliations:** ^1^Department of Pharmacy, The Second Affiliated Hospital of Fujian Medical University, Quanzhou, Fujian, China; ^2^Department of Emergency Medicine, Mindong Hospital Affiliated to Fujian Medical University, Ningde, Fujian, China; ^3^Department of Pharmacology, School of Pharmacy, Fujian Medical University, Fuzhou, China; ^4^Fujian Key Laboratory of Natural Medicine Pharmacology, Fujian Medical University, Fuzhou, China; ^5^Department of Pharmacy, Mindong Hospital Affiliated to Fujian Medical University, Ningde, Fujian, China

**Keywords:** sugemalimab, cost-effectiveness, first-line treatment, gastric or gastroesophageal junction adenocarcinoma, PD-L1

## Abstract

**Background:**

Results from the GEMSTONE-303 trial indicate that compared with placebo plus capecitabine and oxaliplatin (PLA-CAP), sugemalimab plus capecitabine and oxaliplatin (SUG-CAP) as first-line therapy provides clinical benefits for patients with advanced gastric or gastroesophageal junction (G/GEJ) adenocarcinoma with programmed cell death ligand 1 (PD-L1) combined positive score (CPS) ≥5. However, the addition of sugemalimab increases medical costs. This study aimed to assess the cost-effectiveness of SUG-CAP vs. PLA-CAP for the first-line treatment of advanced G/GEJ adenocarcinoma with PD-L1 CPS ≥5 from the perspective of China's healthcare system.

**Methods:**

A Markov model with three health states was developed to compare the cost-effectiveness of SUG-CAP and PLA-CAP. Clinical data were obtained from the GEMSTONE-303 trial, drug costs were determined based on national bidding prices, and other costs and utility values were obtained from published literature. Outcomes included total costs, quality-adjusted life years (QALYs), and incremental cost-effectiveness ratios (ICERs). Sensitivity analysis was used to verify the robustness of the model.

**Results:**

The SUG-CAP incurred costs of $70,673.28 and gained 1.28 QALYs. In the PLA-CAP, the effectiveness was 1.00 QALYs at a cost of $11,241.52. Compared with PLA-CAP, SUG-CAP yielded an increase of 0.28 QALYs at an incremental cost of $59,431.76. The ICER for SUG-CAP vs. PLA-CAP was $217,686.71 per QALY, which exceeds the preset willingness-to-pay (WTP) threshold of $41,511 per QALY, with a 0% probability of being cost-effective. The parameters that significantly affected the model were the cost of sugemalimab, progression-free survival (PFS) utility, and discount rate.

**Conclusion:**

From the perspective of China's healthcare system, SUG-CAP as first-line therapy for advanced G/GEJ adenocarcinoma with PD-L1 CPS ≥5 is not cost-effective compared with chemotherapy alone.

## 1 Introduction

Gastric or gastroesophageal junction (G/GEJ) cancers pose a significant threat to human health, ranking fifth in terms of incidence and mortality rates among malignant tumors (1). In China, the incidence of G/GEJ cancers is notably higher than the global average incidence (1). More than 90% of patients with G/GEJ cancer are adenocarcinoma (2). Owing to the lack of distinct clinical symptoms, most patients are diagnosed at an advanced stage, with a poor prognosis and a 5-year survival rate of < 10% (3). Platinum-based combination chemotherapy is the standard first-line treatment for advanced G/GEJ adenocarcinoma; however, it has limited efficacy, with a median overall survival (OS) of only ~1 year (4–6).

Recent clinical trials have demonstrated that the combination of immune checkpoint inhibitors (ICIs) with chemotherapy can significantly improve survival in patients with advanced G/GEJ adenocarcinoma (6–9). However, from the perspective of China's healthcare system, unlike sintilimab (10), other ICIs, such as pembrolizumab, nivolumab, and tislelizumab in combination with chemotherapy, are not cost-effective for the treatment of advanced G/GEJ adenocarcinoma when compared with chemotherapy alone (11–13). Sugemalimab, an ICI targeting programmed cell death ligand 1 (PD-L1) and a full-length human IgG4 monoclonal antibody, has shown promise in the treatment of G/GEJ adenocarcinoma with PD-L1 combined positive score (CPS) ≥5 (14). The GEMSTONE-303 trial assessed the efficacy and safety of sugemalimab plus capecitabine and oxaliplatin (SUG-CAP) as a first-line treatment for advanced G/GEJ adenocarcinoma with PD-L1 CPS ≥5 (15). The results indicated that compared with placebo plus capecitabine and oxaliplatin (PLA-CAP), SUG-CAP significantly extended the median OS (15.6 vs. 12.6 months), and progression-free survival (PFS; 7.6 vs. 6.1 months), with a similar incidence of adverse events.

Although SUG-CAP has demonstrated clinical efficacy in advanced G/GEJ adenocarcinoma with PD-L1 CPS ≥5, its cost is substantially higher than that of PLA-CAP, which inevitably increases medical expenses and imposes a heavy economic burden on patients and society. This issue is particularly pronounced in countries with limited healthcare resources, such as China. Therefore, evaluating the cost-effectiveness of SUG-CAP is crucial. To the best of our knowledge, no studies have assessed the cost-effectiveness of SUG-CAP as a first-line treatment for advanced G/GEJ adenocarcinoma with PD-L1 CPS ≥5. This study aimed to evaluate the cost-effectiveness of SUG-CAP from the perspective of China's healthcare system, providing a scientific basis for clinical decision-making and healthcare resource allocation.

## 2 Methods

This study was conducted following the Consolidated Health Economic Evaluation Reporting Standards 2022 (Supplementary Table S1) (16).

### 2.1 Model construction

**Figure 1:**
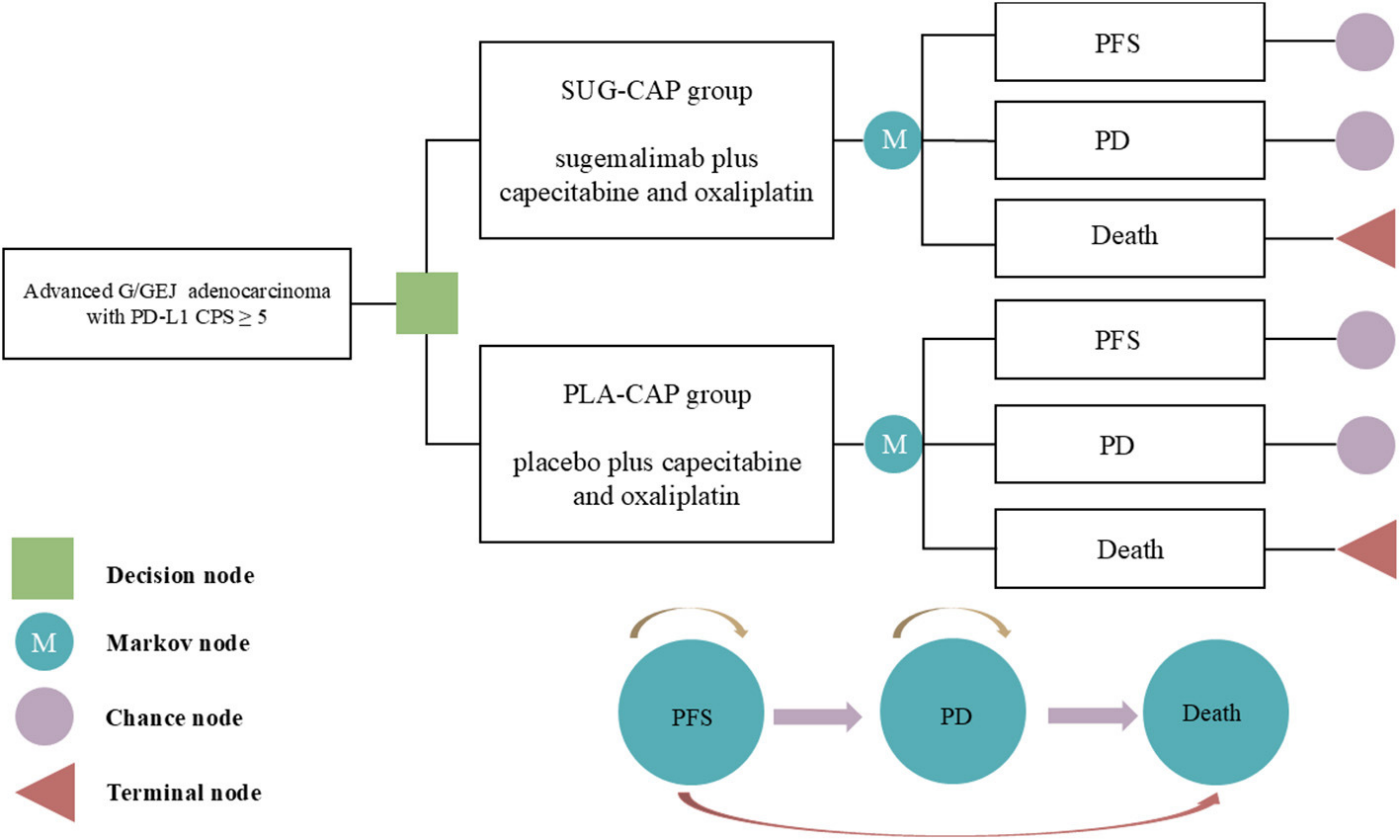
The Markov model simulating outcomes for the GEMSTONE-303 trial. All patients started with PFS state and received treatment with SUG-CAP or PLA-CAP. CPS, combined positive score; G/GEJ, gastric or gastroesophageal junction; PD, progressive disease; PD-L1, programmed death-ligand 1; PFS, progression-free survival; PLA-CAP, placebo plus capecitabine and oxaliplatin; SUG-CAP, sugemalimab plus capecitabine and oxaliplatin.

The TreeAge Pro 2022 software was used to construct a Markov model incorporating three health states, namely, PFS, progressive disease (PD), and death, to evaluate the cost-effectiveness of SUG-CAP vs. PLA-CAP for the first-line treatment of advanced G/GEJ adenocarcinoma patients with PD-L1 CPS ≥5 (Figure 1). All patients entered the model in the PFS state, with death designated as the terminal state. As the model ran, patients could either remain in their current state or transition to the next state but could not return to previous states (17). Based on the treatment schedule from the GEMSTONE-303 trial, each cycle of the model was set at 21 days. The model ran for 200 cycles (~11.6 years), by which time 99% of the patients had died. The outcomes included total costs, quality-adjusted life years (QALYs), and the incremental cost-effectiveness ratio (ICER). According to the China Guidelines for Pharmacoeconomic Evaluation, we established the willingness-to-pay (WTP) threshold at three times China's 2024 per-capita GDP ($41,511 per QALY) (18). A therapeutic strategy was considered cost-effective if its ICER fell below this threshold.

### 2.2 Clinical data

The treatment regimens and outcomes for patients in this study were derived from the GEMSTONE-303 trial (15), a phase 3 randomized controlled study conducted in China. The patients enrolled in the trial had the following characteristics: unresectable locally advanced or metastatic G/GEJ adenocarcinoma, 18–75 years of age, no previous systemic treatment, and PD-L1 CPS ≥5. After enrollment, the patients were randomly assigned to either the SUG-CAP or the PLA-CAP group. Every 21 days is a cycle. In the SUG-CAP group, sugemalimab was administered intravenously at a dose of 1,200 mg on day 1 of each cycle for a maximum duration of 24 months. In both groups, capecitabine was administered orally at a dose of 1,000 mg/m^2^ per administration twice daily on days 1–14 of each cycle, and oxaliplatin was administered intravenously at a dose of 130 mg/m^2^ on day 1 of each cycle. Up to six cycles of treatment. Treatment in both groups continued until PD or unacceptable toxicity. In the trial, the median treatment duration was 6.3 months in the SUG-CAP group and 5.6 months in the PLA-CAP group. Because the trial did not provide detailed data on post-progression treatments, we assumed that all patients received the best supportive care after PD.

### 2.3 Survival transition probabilities

Kaplan–Meier curves for OS and PFS from the GEMSTONE-303 trial were digitized using GetData Graph Digitizer (version 2.26). As described by Guyot et al. (19), the data points were used to reconstruct the survival curves, which were fitted to the following distributions: exponential, Weibull, log-normal, and log-logistic (20, 21). The best-fitting distributions were evaluated using the Akaike Information Criterion and Bayesian Information Criterion (Supplementary Table S2) (22, 23). Eventually, the log-logistic distribution (Table 1) was selected to fit the PFS and OS curves of both SUG-CAP and PLA-CAP groups, and the transition probabilities among the three health states in the model were determined (Supplementary Figure S1). To verify the rationality of the log-logistic distribution used in our model, spline-based approaches were employed. The results indicated that the health state probabilities estimated by these two methods were generally in agreement, as shown in Supplementary Table S3.

**Table 1:** Relevant parameters of survival distribution.

**Variable**	**Value**	**Source**
**Log-logistic survival model of PFS**		
SUG-CAP group	Scale = 0.1281212, Shape = 2.018239	(15)
PLA-CAP group	Scale = 0.1749994, Shape = 2.175263	(15)
**Log-logistic survival model of OS**		
SUG-CAP group	Scale = 0.06329585, Shape = 1.794441	(15)
PLA-CAP group	Scale = 0.08067551, Shape = 1.871019	(15)

OS, overall survival; PFS, progression-free survival; PLA-CAP, placebo plus capecitabine and oxaliplatin; SUG-CAP, sugemalimab plus capecitabine and oxaliplatin.

### 2.4 Costs and utilities

This study considered only direct medical costs, which included the costs of drugs, tests, routine follow-ups, the best supportive care, management of adverse reactions with an incidence rate of >5% (grade 3 or above), and terminal care (Table 2). Drug costs were determined based on national bidding prices, whereas other costs were obtained from published literature and adjusted to 2024 values using the Chinese Medical Price Index (24). All costs were converted to US dollars using the 2024 average exchange rate between the Chinese yuan and the US dollar (1 USD = 7.12 CNY). For ease of calculating drug dosages, the body surface area of the patients was assumed to be 1.72 m^2^ (25). Health utility values ranging from 0 (death) to 1 (perfect health) were used to assess the three health states, namely, PFS, PD, and death. Because the GEMSTONE-303 trial did not report health utility values, we extracted these values from a previous Chinese study (25), which is a cost-effectiveness analysis of tislelizumab as first-line therapy for advanced G/GEJ adenocarcinoma from China's healthcare system perspective. The analyzed population is highly consistent with this study. And considered the disutility values associated with adverse reactions to minimize bias. All costs and utilities were discounted at a rate of 5% (18).

**Table 2:** Basic parameters of the model and the range of sensitivity analysis.

**Parameter**	**Base value**	**Range**		**Distribution**	**Source**
		**Min**	**Max**		
**Risk of adverse events**					
**SUG-CAP group**					
Decreased white blood cell count	0.066	0.053	0.079	Beta	(15)
Anemia	0.108	0.086	0.130	Beta	(15)
Decreased neutrophil count	0.141	0.113	0.169	Beta	(15)
Decreased platelet count	0.183	0.146	0.220	Beta	(15)
**PLA-CAP group**					
Decreased white blood cell count	0.030	0.024	0.036	Beta	(15)
Anemia	0.072	0.058	0.086	Beta	(15)
Decreased neutrophil count	0.143	0.114	0.172	Beta	(15)
Decreased platelet count	0.160	0.128	0.192	Beta	(15)
**Cost ($)**					
Sugemalimab (600 mg)	1,738.06	1,390.45	2,085.67	Gamma	(38)
Oxaliplatin (100 mg)	32.88	26.30	39.46	Gamma	(38)
Capecitabine (500 mg)	0.75	0.60	0.90	Gamma	(38)
Decreased white blood cell count	466.93	373.55	560.32	Gamma	(21)
Anemia	532.76	426.21	639.32	Gamma	(21)
Decreased neutrophil count	462.42	369.94	554.91	Gamma	(21)
Decreased platelet count	1,056.33	845.06	1,267.59	Gamma	(39)
Terminal care	1,463.22	1,170.58	1,755.86	Gamma	(21)
Best supportive care per cycle	164.90	131.92	197.88	Gamma	(25)
Routine follow-up per cycle	80.87	64.70	97.05	Gamma	(25)
Tests per cycle	357.70	286.16	429.24	Gamma	(40)
**Utility**					
PFS	0.797	0.638	0.956	Beta	(25)
PD	0.577	0.462	0.692	Beta	(25)
**Dis-utility of adverse events**					
Decreased white blood cell count	0.200	0.160	0.240	Beta	(41)
Anemia	0.073	0.058	0.088	Beta	(41)
Decreased neutrophil count	0.200	0.160	0.240	Beta	(41)
Decreased platelet count	0.023	0.018	0.028	Beta	(41)
Discount rate	0.05	0	0.08	Fixed	(18)
Body surface area (m^2^)	1.72	1.38	2.06	Normal	(25)

PD, progressive disease; PFS, progression-free survival; PLA-CAP, placebo plus capecitabine and oxaliplatin; SUG-CAP, sugemalimab plus capecitabine and oxaliplatin.

### 2.5 Sensitivity analysis

We performed one-way and probabilistic sensitivity analyses. One-way sensitivity analysis was used to assess how the results of the model were affected by changes in individual parameters within certain ranges. All parameters were adjusted within the 95% confidence intervals reported in the literature or by ±20% of the baseline value when data were unavailable. The discount rate ranged from 0 to 8% (Table 2), and the results were presented on tornado plots. Probabilistic sensitivity analysis was used to evaluate the impact of simultaneous changes in all parameters on the results of the model. In 1,000 Monte Carlo simulation iterations, all parameters were randomly altered based on pre-specified distributions (Table 2), and the results were visualized on scatter plots and cost-effectiveness acceptability curve. In line with the ISPOR-SMDM Modeling Good Research Practices Task Force Working Group6′s recommendation (26), we adopted a gamma distribution for cost modeling, a normal distribution for body surface area modeling, and a beta distribution for adverse event incidence and utility value modeling. In addition, we have also explored the price of sugemalimab at which SUG-CAP is cost-effective by gradually reducing the price of sugemalimab.

### 2.6 Scenario analysis

In scenario 1, because post-follow-up survival was determined based on fitted data, we set the model run time to the follow-up period of the clinical trial (2.09 years) to assess its impact on the results. In scenario 2, we assumed that only 30 or 50% of the patients received the best supportive care after disease progression, simulating real-world situations where some patients discontinue treatment for various reasons. Scenario 3: although the WTP threshold in this study was set at three times China's per capita GDP, in line with the recommendation of the China Guidelines for Pharmacoeconomic Evaluation (18), Cai et al. (27) argued that Chinese medical insurance policy-makers, with their strong bargaining power, often prefer a lower threshold. They suggest 1.5 times the per capita GDP as a reference threshold for medical insurance decision-makers to minimize sub-optimal decisions. Thus, we adjusted the WTP threshold to 1.5 times China's per capita GDP ($20,756/QALY) to assess SUG-CAP's cost-effectiveness. Scenario 4: we adjusted sugemalimab's price to 50%, 20%, and 10% of its current price to explore SUG-CAP's cost-effectiveness under different pricing scenarios.

### 2.7 Subgroup analysis

Exploratory subgroup analysis was performed to assess the effects of different baseline characteristics of the patients on model outcomes. The subgroups were stratified based on age, sex, the Eastern Cooperative Oncology Group performance status, primary tumor location, organs with metastasis, liver metastasis, tumor stage at screening, previous treatment, and PD-L1 expression (Table 3). Owing to insufficient survival data, we used the same PFS and OS functions (log-logistic survival model) for all subgroups in the PLA-CAP arm as for the overall population. According to a method described by Hoyle et al. (28) and the subgroup-specific hazard ratios obtained from the GEMSTONE-303 trial, the ICER and cost-effectiveness acceptability probability were calculated for each subgroup.

**Table 3:** Results of subgroup analyses.

**Subgroup**	**OS HR (95% CI)**	**PFS HR (95% CI)**	**ICER ($/QALY)**
**Age, year**			
< 65	0.80 (0.61–1.06)	0.71 (0.54–0.93)	174,090.52
≥65	0.68 (0.50–0.93)	0.61 (0.45–0.83)	124,447.92
**Sex**			
Male	0.79 (0.62–1.00)	0.65 (0.51–0.83)	156,582.66
Female	0.65 (0.44–0.98)	0.70 (0.48–1.03)	124,412.72
**Baseline ECOG performance status**			
0	0.70 (0.45–1.09)	0.55 (0.36–0.84)	122,626.85
1	0.76 (0.60–0.96)	0.70 (0.55–0.88)	157,653.48
**Primary tumor location**			
Stomach	0.76 (0.61–0.94)	0.67 (0.54–0.83)	152,013.89
Gastroesophageal junction	0.59 (0.29–1.20)	0.50 (0.25–1.00)	102,613.71
**No. of organs with metastasis**			
1–2	0.73 (0.55–0.97)	0.69 (0.52–0.92)	146,416.37
≥3	0.80 (0.58–1.12)	0.66 (0.48–0.91)	161,642.71
**Liver metastases**			
Yes	0.57 (0.41–0.79)	0.55 (0.40–0.76)	100,893.94
No	0.85 (0.65–1.12)	0.72 (0.55–0.94)	197,711.02
**Tumor stage at screening**			
III	0.43 (0.14–1.29)	0.33 (0.11–1.01)	83,909.17
IV	0.76 (0.62–0.94)	0.68 (0.56–0.84)	153,852.37
**Prior treatment**			
Yes	0.62 (0.38–1.01)	0.62 (0.38–0.99)	112,551.23
No	0.79 (0.63–0.99)	0.68 (0.54–0.85)	163,200.15
**PD-L1 expression (CPS), %**			
5–9	0.88 (0.65–1.19)	0.78 (0.58–1.04)	244,243.46
≥10	0.65 (0.49–0.86)	0.58 (0.44–0.77)	116,193.19

CI, confidence interval; CPS, combined positive score; ECOG, the Eastern Cooperative Oncology Group; HR, hazard ratio; ICER, incremental cost-effectiveness ratio; OS, overall survival; PD-L1, programmed death-ligand 1; PFS, progression-free survival.

## 3 Results

### 3.1 Base-case analysis

The SUG-CAP group generated 1.28 QALYs at a cost of $70,673.28, while the PLA-CAP group generated 1.00 QALYs at a cost of $11,241.52. SUG-CAP achieved an incremental effect of 0.28 QALYs at an extra cost of $59,431.76 vs. PLA-CAP. As shown in Table 4, the ICER of SUG-CAP vs. PLA-CAP is $217,686.71 per QALY, surpassing the preset WTP threshold of $41,511 per QALY. This suggests that, compared with PLA-CAP, SUG-CAP isn't cost-effective as a first-line treatment for advanced G/GEJ adenocarcinoma with PD-L1 CPS ≥5.

**Table 4:** Costs and outcomes of the cost-effectiveness analysis.

**Regimen**	**SUG-CAP group**	**PLA-CAP group**	**Increment**
Total QALYs	1.28	1.00	0.28
Total cost, $	70,673.28	11,241.52	59,431.76
ICER, $ per QALY			217,686.71

ICER, incremental cost-effectiveness ratio; PLA-CAP, placebo plus capecitabine and oxaliplatin; QALY, quality-adjusted life year; SUG-CAP, sugemalimab plus capecitabine and oxaliplatin.

### 3.2 Sensitivity analysis

**Figure 2:**
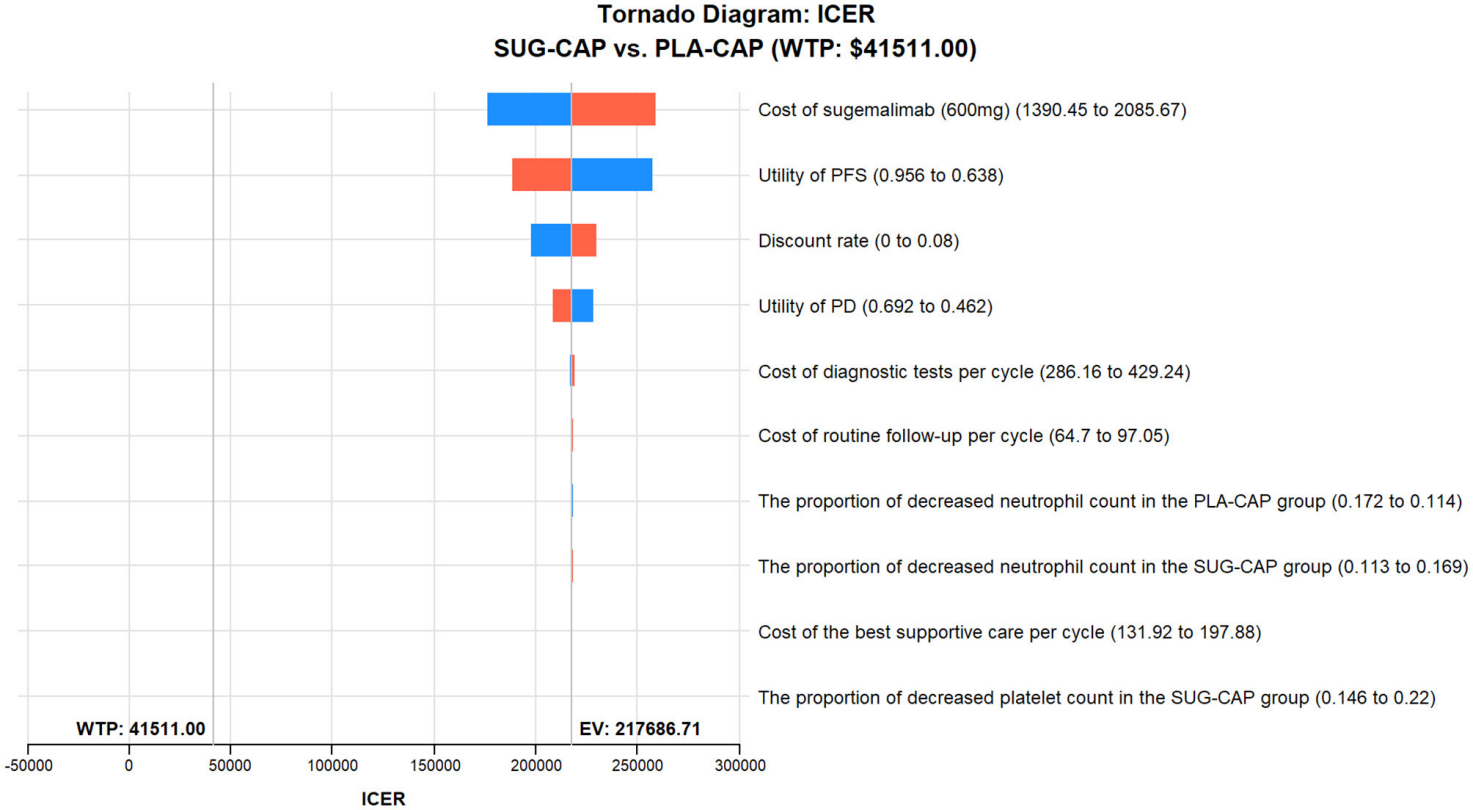
One-way sensitivity analyses comparing the SUG-CAP and PLA-CAP groups. ICER, incremental cost-effectiveness ratio; PD, progressive disease; PFS, progression-free survival; PLA-CAP, placebo plus capecitabine and oxaliplatin; SUG-CAP, sugemalimab plus capecitabine and oxaliplatin; WTP, willingness-to-pay.

**Figure 3:**
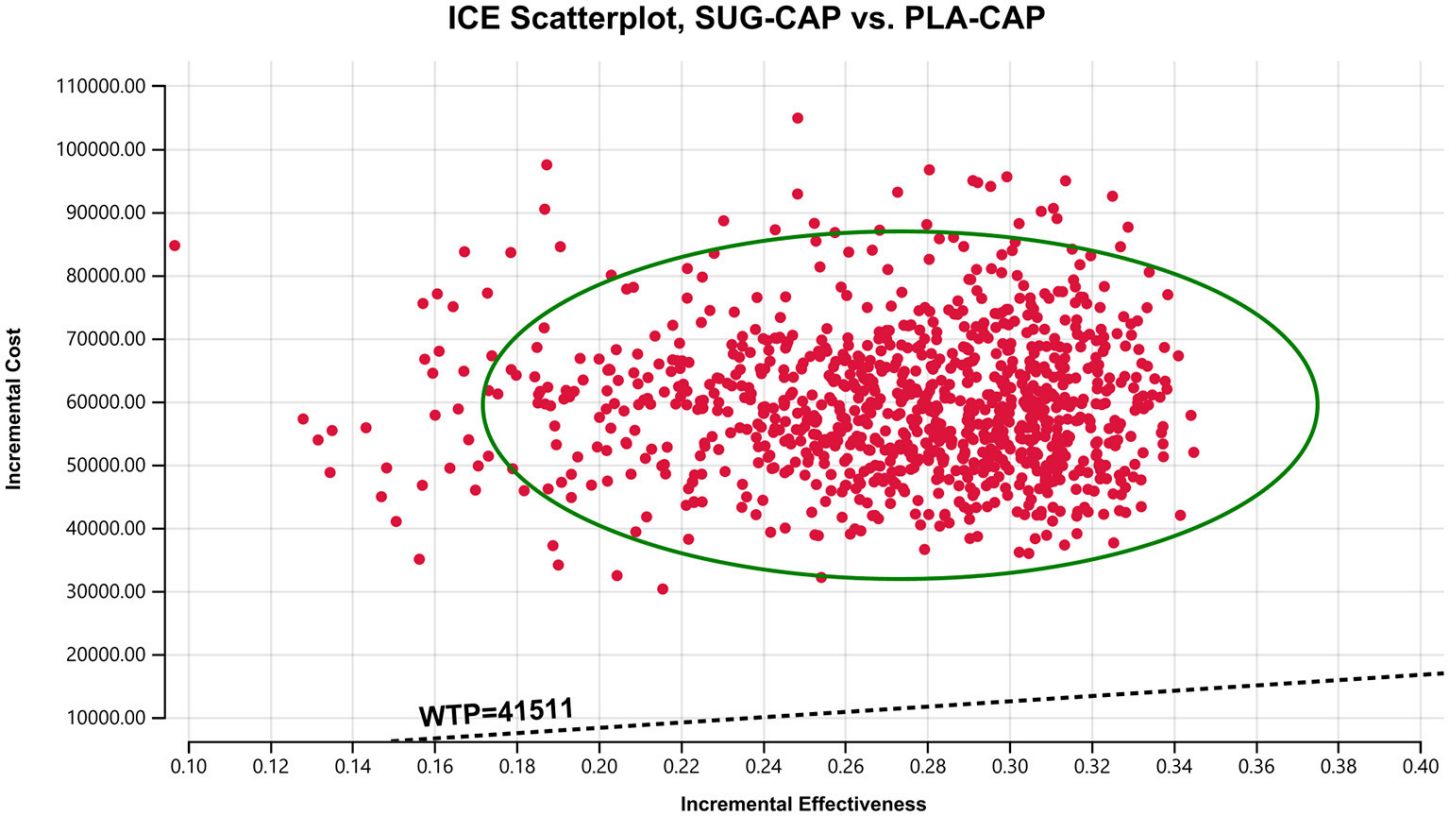
A probabilistic scatter plot of the ICER between the SUG-CAP group and the PLA-CAP group. ICE, incremental cost-effectiveness; PLA-CAP, placebo plus capecitabine and oxaliplatin; SUG-CAP, sugemalimab plus capecitabine and oxaliplatin; WTP, willingness-to-pay.

**Figure 4:**
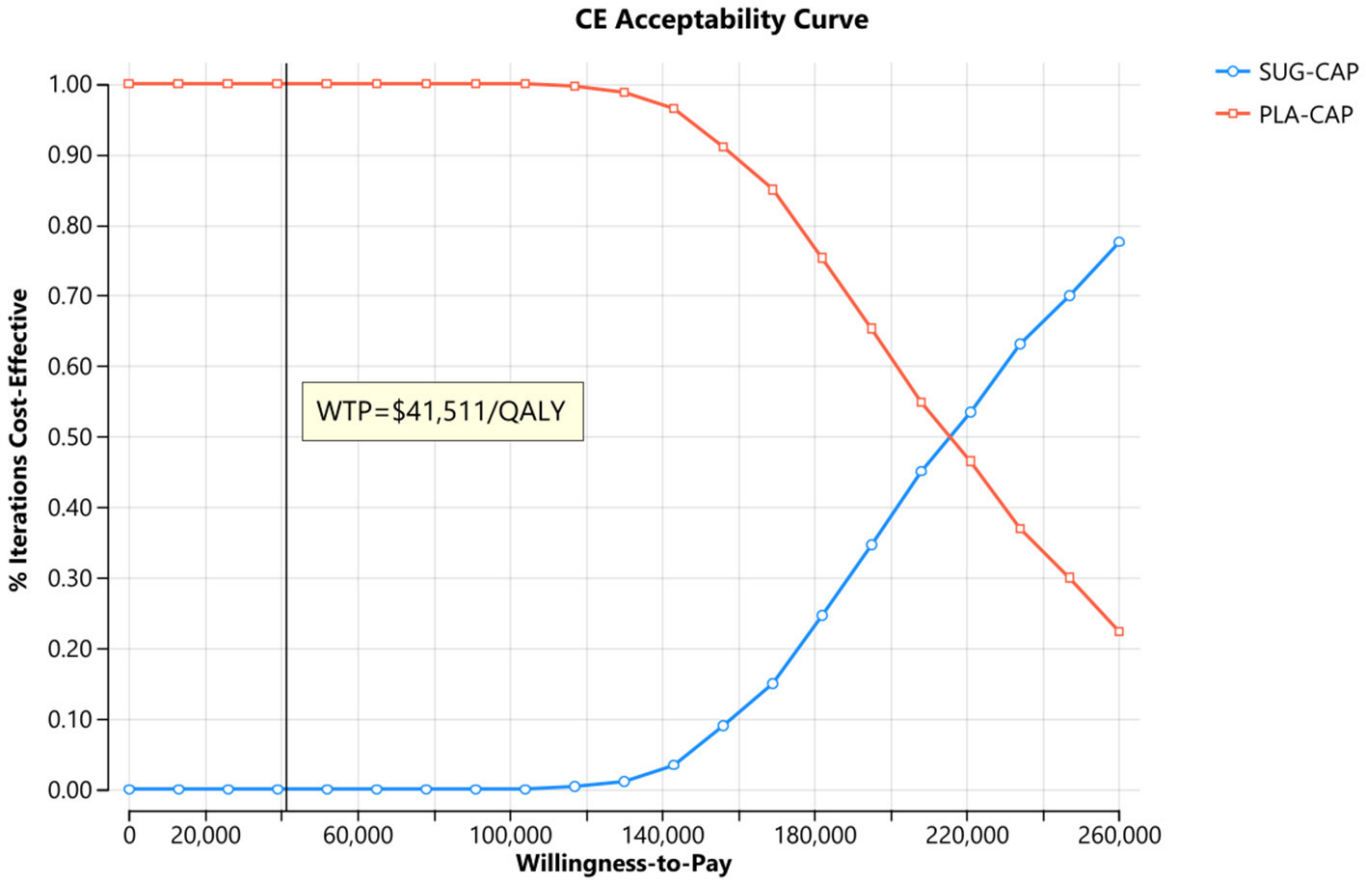
The cost-effectiveness acceptability curves for the SUG-CAP group compared with the PLA-CAP group. CE, cost-effectiveness; PLA-CAP, placebo plus capecitabine and oxaliplatin; SUG-CAP, sugemalimab plus capecitabine and oxaliplatin; WTP, willingness-to-pay.

As depicted in the tornado diagram (Figure 2), the one-way sensitivity analysis indicates that parameters such as the cost of sugemalimab, PFS utility, and discount rate influence the model. Nevertheless, altering these parameters within given ranges still leaves the ICER above the preset WTP threshold, implying their limited impact on the model results. The results of probabilistic sensitivity analysis are shown in the scatter plot (Figure 3) and cost-effectiveness acceptability curve (Figure 4). When the WTP threshold was $41,511/QALY, the probability of SUG-CAP being cost-effective compared with PLA-CAP for the first-line treatment of advanced G/GEJ adenocarcinoma with PD-L1 CPS ≥5 was 0%. SUG-CAP could become a cost-effective treatment strategy compared with PLA-CAP only when the price of sugemalimab decreases below $267.4.

### 3.3 Scenario analysis

Table 5 shows the results of the scenario analysis. In scenario 1, when the model duration was 2.09 years, the ICER of SUG-CAP vs. PLA-CAP was $391,793.01 per QALY, which indicated the cost-ineffectiveness of SUG-CAP. In scenario 2, when the proportion of patients receiving the best supportive care was 30% and 50%, the ICERs of SUG-CAP vs. PLA-CAP were $216,479.55 per QALY and $216,824.45 per QALY, respectively, indicating minimal changes in ICER values. In scenario 3, when the WTP threshold was set at 1.5 times China's per capita GDP, SUG-CAP was still not cost-effective compared with PLA-CAP. In scenario 4, even when the price of sugemalimab was reduced to 50% or 30% of the current price, SUG-CAP remained not cost-effective. Only when the price was reduced to 10% did SUG-CAP become cost-effective.

**Table 5:** Results of scenario analysis.

**Scenarios**	**Cost ($)**		**QALYs**		**ICER ($/QALY)**
	**SUG-CAP group**	**PLA-CAP group**	**SUG-CAP group**	**PLA-CAP group**	
Scenario 1	60,202.36	9,530.16	0.91	0.78	391,793.01
**Scenario 2**					
30% of patients received the best supportive care	67,929.63	8,827.45	1.28	1.00	216,479.55
50% of patients received the best supportive care	68,713.53	9,517.18	1.28	1.00	216,824.45
**Scenario 4**					
50% of the current price of sugemalimab	42,249.88	11,241.52	1.28	1.00	113,577.46
20% of the current price of sugemalimab	25,195.84	11,241.52	1.28	1.00	51,111.90
10% of the current price of sugemalimab	19,511.16	11,241.52	1.28	1.00	30,290.05

Scenario 1 = model truncated to trial follow-up duration; Scenario 2 = adjust the proportion of patients receiving the best supportive care after disease progression; Scenario 4 = adjust the price of sugemalimab.

ICER, incremental cost-effectiveness ratio; PLA-CAP, placebo plus capecitabine and oxaliplatin; QALY, quality-adjusted life year; SUG-CAP, sugemalimab plus capecitabine and oxaliplatin.

### 3.4 Subgroup analysis

In all subgroups, the ICER of SUG-CAP vs. PLA-CAP exceeded the WTP threshold of $41,511 per QALY, with a 0% probability of cost-effectiveness. Notably, the ICER was relatively low in patients with tumor stage III at screening, liver metastases, and primary GEJ cancer (Table 3). However, owing to the small sample size in these subgroups, the results should be interpreted with caution.

## 4 Discussion

In the GEMSTONE-303 trial, compared with PLA-CAP, SUG-CAP as a first-line treatment showed clinical efficacy for advanced G/GEJ adenocarcinoma with PD-L1 CPS ≥5, extending the median OS by 3 months and median PFS by 1.5 months and exhibiting good tolerability. These findings highlighted the potential of SUG-CAP as a new first-line treatment for patients of advanced G/GEJ adenocarcinoma with PD-L1 CPS ≥5. However, adding sugemalimab to a chemotherapy regimen increases costs, challenging healthcare system sustainability. Therefore, assessing the cost-effectiveness of SUG-CAP is essential. In this study, we evaluated the cost-effectiveness of SUG-CAP vs. PLA-CAP for the first-line treatment of advanced G/GEJ cancer with PD-L1 CPS ≥5 from the perspective of China's healthcare system. The findings of this study offer crucial economic insights into the Chinese and global healthcare systems.

Base-case analysis showed that the ICER of SUG-CAP vs. PLA-CAP was $217,686.71 per QALY, which exceeded the WTP threshold of $41,511 per QALY. Sensitivity analysis indicated near-0% cost-effectiveness of SUG-CAP at this threshold. These results indicate that from the perspective of China's healthcare system, SUG-CAP is not a cost-effective therapy compared with PLA-CAP for advanced G/GEJ cancer with PD-L1 CPS ≥5. The likely reason for this cost-ineffectiveness is that sugemalimab does not provide a sufficient incremental survival benefit despite being more costly than capecitabine and oxaliplatin. In one cycle of treatment, SUG-CAP had 24.85 times the drug cost of PLA-CAP but only a 28% increase in total QALYs. The results of one-way sensitivity analysis showed that the cost of sugemalimab was the most important factor influencing the cost-effectiveness of SUG-CAP, which validates the above inference. Therefore, reducing the price of sugemalimab is particularly important to improve access to the SUG-CAP regimen for patients with advanced G/GEJ cancer with PD-L1 CPS ≥5. Since its establishment in 2018, China's National Healthcare Security Administration has conducted multiple rounds of drug price negotiations with pharmaceutical companies through the national procurement strategy. Consequently, the prices of many anti-cancer drugs have decreased by 30%−70%, significantly reducing the economic burden on patients with cancer (29). Sugemalimab was approved in China at the end of 2021 but has not yet been included in national medical insurance price negotiations. Therefore, we believe the price of sugemalimab can be substantially reduced if it enters the negotiation process. This study indicates that SUG-CAP may become a cost-effective treatment option when the price of sugemalimab is reduced to below $267.4. This finding offers a valuable reference for price negotiations of sugemalimab in China.

One-way sensitivity analysis showed that the cost of sugemalimab, utility value of PFS, and discount rate had the most significant effect on the model. However, altering these parameters within pre-determined ranges did not affect model outcomes. Probabilistic sensitivity analysis showed that compared with PLA-CAP, SUG-CAP had a 0% chance of being cost-effective for advanced G/GEJ cancer with a PD-L1 CPS of ≥5. These results validated the robustness of the model.

In scenario 1, SUG-CAP remained cost-ineffective when the model run time was set to the follow-up period of the GEMSTONE-303 trial (2.09 years), indicating that the run time of the model beyond the trial follow-up time had no effect on the results. In scenario 2, the ICER of SUG-CAP vs. PLA-CAP showed a minimal change when the number of patients receiving treatment after disease progression varied. This finding suggests that undergoing treatment after disease progression does not decrease the cost-effectiveness of SUG-CAP. However, these results may be well-accepted by doctors and patients, as supporting continued treatment after disease progression is consistent with ethical and moral standards. The findings from the analyses of scenarios 3 and 4 provide useful economic guidance for future health insurance price negotiations for sugemalimab. Furthermore, exploratory subgroup analysis showed that although the SUG-CAP regimen was not cost-effective in any subgroup, its ICER values were relatively low in patients with tumor stage III at screening, liver metastases, and primarily GEJ cancer, indicating the relatively high cost-effectiveness of SUG-CAP in these subgroups. These findings indicate that designing individualized treatment regimens can enhance the cost-effectiveness of SUG-CAP.

To date, eight studies have evaluated the cost-effectiveness of sugemalimab for cancer treatment from the perspective of China's healthcare system. Cai et al. (30) found that compared with chemotherapy, sugemalimab combined with chemotherapy was not cost-effective for patients with advanced esophageal squamous cell carcinoma. Cheng et al. (31) reported that sugemalimab plus chemotherapy as a first-line treatment for metastatic squamous or non-squamous non-small cell lung cancer (NSCLC) was not cost-effective compared with chemotherapy alone. Li et al. (32) showed that compared with placebo, sugemalimab consolidation therapy was not cost-effective for patients with unresectable stage-III NSCLC receiving chemoradiotherapy. Liang et al. (33), Wang et al. (34), Chen et al. (35), Li et al. (36), and Zheng et al. (37) concluded that sugemalimab combined with chemotherapy was not cost-effective compared with chemotherapy for patients with metastatic NSCLC. These findings are consistent with those of our study.

This study has several strengths. First, all patients in the GEMSTONE-303 trial were Chinese, which allowed the direct assessment of the cost and clinical efficacy of SUG-CAP in China's healthcare system. Consequently, the results of this study can be generalized to the Chinese population. Second, our extensive subgroup and scenario analyses revealed the impact of SUG-CAP across diverse patient groups and treatment settings, offering valuable guidance for developing individualized treatment strategies and medical insurance policies. Finally, the findings of this study provide crucial economic evidence for national medical insurance price negotiations for sugemalimab.

Despite notable strengths, this study has several limitations that should be noted. First, because the GEMSTONE-303 trial did not provide health utility values, we extracted the utility values from another Chinese study, which might have led to bias in the results. However, the sensitivity analysis showed that this does not affect the model's results. In the future, we will update our research when health utility values for the Chinese population become available. Second, we considered the disutility and treatment costs of only severe adverse events with an incidence of ≥5%, which might have affected the total cost and QALYs in the model. Third, owing to the ongoing nature of the GEMSTONE-303 trial, long-term patient survival data were unavailable. We used survival models to extrapolate data beyond the follow-up period, which may be different from actual data. For example, the survival curves of patients receiving immunotherapy may plateau in the tail end. Our model does not account for the possibility of long-term survival and may, thus underestimate the efficacy of immunotherapy. Future studies should validate these findings using real-world data for cost-effectiveness analysis. Fourth, the trial did not provide detailed information regarding treatment after the failure of the first-line treatment. Consequently, we assumed that all patients received optimal supportive care after disease progression, which might not have adequately reflected real-world clinical cases. In reality, the choice of subsequent treatment regimens is individually determined based on each patient's specific circumstances. Fortunately, the results of the one-way sensitivity analysis and scenario analysis are reassuring, as they consistently indicate that changing the range of subsequent-line treatment estimates does not alter the model outcomes. Finally, the relatively small sample size in the subgroup analyses may compromise statistical power and result stability. Additionally, the wide confidence intervals of subgroups reflect some uncertainty in the findings. Thus, the subgroup results should be interpreted cautiously to avoid overgeneralization.

## 5 Conclusion

From the perspective of China's healthcare system, SUG-CAP as a first-line treatment for advanced G/GEJ adenocarcinoma with PD-L1 CPS ≥5 may not be cost-effective compared with chemotherapy alone.

## Data Availability

The original contributions presented in the study are included in the article/Supplementary material, further inquiries can be directed to the corresponding author.
