# Supplementary material for: Cost-effective analysis of sugemalimab plus chemotherapy as first-line treatment for advanced gastric or gastroesophageal junction adenocarcinoma with PD-L1 CPS ≥5
Source: Front Public Health. 2025 Aug 22;13:1604372. doi: 10.3389/fpubh.2025.1604372 (PMC12411535; doi:10.3389/fpubh.2025.1604372)
Supplement: Supplementary file 1 [file Data_Sheet_1.docx]

Supplementary Material

**Cost-effective Analysis of Sugemalimab plus Chemotherapy as First-Line Treatment for Advanced Gastric or Gastroesophageal Junction Adenocarcinoma with PD-L1 CPS ≥ 5**

**1. Supplementary Table A. CHEERS 2022 Checklist.**

**2. Supplementary Table B. Comparison of survival models.**

**3.** **Supplementary Figure A. Results of the survival curve fit the SUG-CAP and PLA-CAP group.**

**4. Supplementary Table C. Compare health state probabilities from log-logistic distribution and spline-based approaches**

**1.** **Supplementary Table A. CHEERS 2022 Checklist.**

| **Topic** | **No.** | **Item** | **Reported** |
| --- | --- | --- | --- |
| **Title** |  |  |  |
|  | 1 | Identify the study as an economic evaluation and specify the interventions being compared. | Yes |
| **Abstract** |  |  |  |
|  | 2 | Provide a structured summary that highlights context, key methods, results, and alternative analyses. | Yes |
| **Introduction** |  |  |  |
| **Background and objectives** | 3 | Give the context for the study, the study question, and its practical relevance for decision making in policy or practice. | Yes |
| **Methods** |  |  |  |
| **Health economic analysis plan** | 4 | Indicate whether a health economic analysis plan was developed and where available. | Yes |
| **Study population** | 5 | Describe characteristics of the study population (such as age range, demographics, socioeconomic, or clinical characteristics). | Yes |
| **Setting and location** | 6 | Provide relevant contextual information that may influence findings. | Yes |
| **Comparators** | 7 | Describe the interventions or strategies being compared and why chosen. | Yes |
| **Perspective** | 8 | State the perspective(s) adopted by the study and why chosen. | Yes |
| **Time horizon** | 9 | State the time horizon for the study and why appropriate. | Yes |
| **Discount rate** | 10 | Report the discount rate(s) and reason chosen. | Yes |
| **Selection of outcomes** | 11 | Describe what outcomes were used as the measure(s) of benefit(s) and harm(s). | Yes |
| **Measurement of outcomes** | 12 | Describe how outcomes used to capture benefit(s) and harm(s) were measured. | Yes |
| **Valuation of outcomes** | 13 | Describe the population and methods used to measure and value outcomes. | Yes |
| **Measurement and valuation of resources and costs** | 14 | Describe how costs were valued. | Yes |
| **Currency, price date, and conversion** | 15 | Report the dates of the estimated resource quantities and unit costs, plus the currency and year of conversion. | Yes |
| **Rationale and description of model** | 16 | If modelling is used, describe in detail and why used. Report if the model is publicly available and where it can be accessed. | Yes |
| **Analytics and assumptions** | 17 | Describe any methods for analysing or statistically transforming data, any extrapolation methods, and approaches for validating any model used. | Yes |
| **Characterising heterogeneity** | 18 | Describe any methods used for estimating how the results of the study vary for subgroups. | Yes |
| **Characterising distributional effects** | 19 | Describe how impacts are distributed across different individuals or adjustments made to reflect priority populations. | Yes |
| **Characterising uncertainty** | 20 | Describe methods to characterise any sources of uncertainty in the analysis. | Yes |
| **Approach to engagement with patients and others affected by the study** | 21 | Describe any approaches to engage patients or service recipients, the general public, communities, or stakeholders (such as clinicians or payers) in the design of the study. | Not applicable |
| **Results** |  |  |  |
| **Study parameters** | 22 | Report all analytic inputs (such as values, ranges, references) including uncertainty or distributional assumptions. | Yes |
| **Summary of main results** | 23 | Report the mean values for the main categories of costs and outcomes of interest and summarise them in the most appropriate overall measure. | Yes |
| **Effect of uncertainty** | 24 | Describe how uncertainty about analytic judgments, inputs, or projections affect findings. Report the effect of choice of discount rate and time horizon, if applicable. | Yes |
| **Effect of engagement with patients and others affected by the study** | 25 | Report on any difference patient/service recipient, general public, community, or stakeholder involvement made to the approach or findings of the study | Not applicable |
| **Discussion** |  |  |  |
| **Study findings, limitations, generalisability, and current knowledge** | 26 | Report key findings, limitations, ethical or equity considerations not captured, and how these could affect patients, policy, or practice. | Yes |
| **Other relevant information** |  |  |  |
| **Source of funding** | 27 | Describe how the study was funded and any role of the funder in the identification, design, conduct, and reporting of the analysis | Yes |
| **Conflicts of interest** | 28 | Report authors conflicts of interest according to journal or International Committee of Medical Journal Editors requirements. | Yes |

**2.** **Supplementary Table B. Comparison of survival models.**

|  | AIC | | BIC | |
| --- | --- | --- | --- | --- |
|  | SUG-CAP group | PLA-CAP group | SUG-CAP group | PLA-CAP group |
| OS |  |  |  |  |
| Exponential | 1489.964 | 1554.250 | 1489.954 | 1554.564 |
| Weibull | 1385.166 | 1451.653 | 1392.340 | 1458.753 |
| Log-logistic | 1365.728 | 1429.549 | 1372.647 | 1442.454 |
| Log-normal | 1370.267 | 1439.205 | 1376.919 | 1445.858 |
| PFS |  |  |  |  |
| Exponential | 1222.522 | 1208.936 | 1222.522 | 1212.429 |
| Weibull | 1191.789 | 1177.059 | 1198.933 | 1184.127 |
| Log-logistic | 1162.126 | 1152.730 | 1165.047 | 1159.637 |
| Log-normal | 1163.116 | 1158.267 | 1169.801 | 1164.938 |

AIC, Akaike information criterion; BIC, Bayesian information criterion; OS, overall survival; PFS, progression-free survival; PLA-CAP, placebo plus capecitabine and oxaliplatin; SUG-CAP, sugemalimab plus capecitabine and oxaliplatin.

**3.** **Supplementary** **Figure A. Results of the survival curve fit the SUG-CAP and PLA-CAP group.**

**
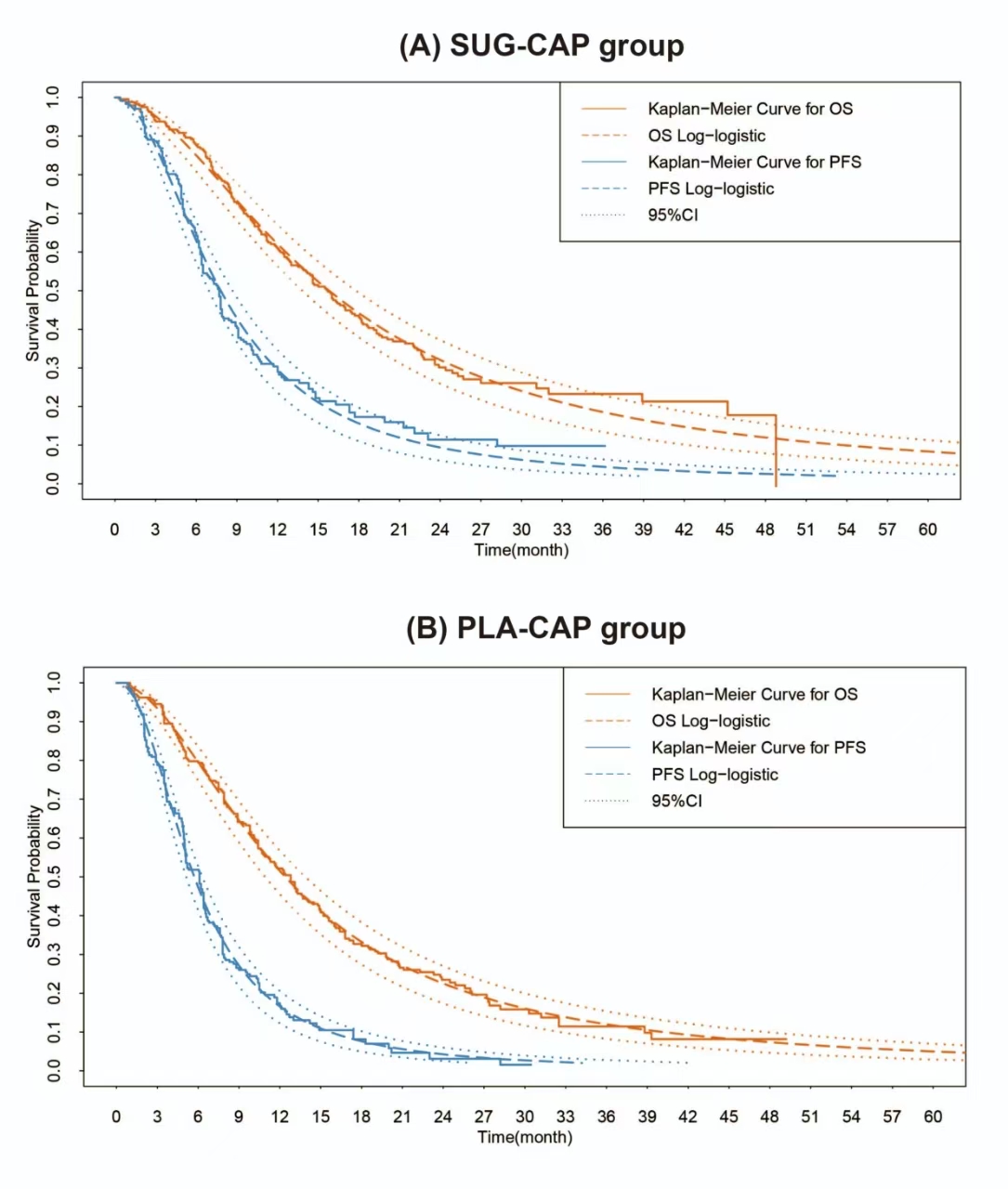
**

95%CI: 95% confidence interval; OS, overall survival; PFS, progression-free survival; PLA-CAP, placebo plus capecitabine and oxaliplatin; SUG-CAP, sugemalimab plus capecitabine and oxaliplatin.

**4. Supplementary Table C. Compare health state probabilities from log-logistic distribution and spline-based approaches.**

| Time (months) | PFS of PLA-CAP group | |  | OS of PLA-CAP group | |  | PFS of SUG-CAP | |  | OS of SUG-CAP | |
| --- | --- | --- | --- | --- | --- | --- | --- | --- | --- | --- | --- |
|  | spline-based approaches | log-logistic distribution |  | spline-based approaches | log-logistic distribution |  | spline-based approaches | log-logistic distribution |  | spline-based approaches | log-logistic distribution |
| 6 | 0.474 | 0.475 |  | 0.789 | 0.791 |  | 0.628 | 0.629 |  | 0.871 | 0.858 |
| 12 | 0.165 | 0.166 |  | 0.515 | 0.514 |  | 0.291 | 0.295 |  | 0.606 | 0.623 |
| 18 | 0.083 | 0.076 |  | 0.324 | 0.332 |  | 0.181 | 0.157 |  | 0.426 | 0.445 |
| 24 | 0.031 | 0.028 |  | 0.237 | 0.225 |  | 0.117 | 0.093 |  | 0.305 | 0.323 |
| 30 | 0.015 | 0.018 |  | 0.152 | 0.161 |  | 0.093 | 0.062 |  | 0.257 | 0.248 |

OS, overall survival; PFS, progression-free survival; PLA-CAP, placebo plus capecitabine and oxaliplatin; SUG-CAP, sugemalimab plus capecitabine and oxaliplatin.
